# Intraoperative and fluorescein angiographic findings of a secondary macular hole associated with age-related macular degeneration treated by pars plana vitrectomy

**DOI:** 10.1186/1471-2415-14-114

**Published:** 2014-09-30

**Authors:** Tomohiro Okamoto, Hajime Shinoda, Toshihide Kurihara, Norihiro Nagai, Kazuo Tsubota, Yoko Ozawa

**Affiliations:** Laboratory of Retinal Cell Biology, Keio University School of Medicine, 35 Shinanomachi, Shinjuku-ku, Tokyo 160-8582 Japan; Department of Ophthalmology, Keio University School of Medicine, 35 Shinanomachi, Shinjuku-ku, Tokyo 160-8582 Japan

**Keywords:** Age-related macular degeneration, Vitreo-retinal interface, Macular hole

## Abstract

**Background:**

Macular hole results from a tractional force at the vitreo-retinal interface which is developed by modification and subsequent degeneration of the posterior precortical vitreous and the internal limiting membrane (ILM). Meanwhile, the wet type of age-related macular degeneration (AMD) is caused by the submacular formation of choroidal neovascularization (CNV). Although exudative changes derived from CNV may cause epiretinal membrane (ERM) formation, which can also cause tractional force at the vitreo-retinal interface, there have been few reports of AMD-associated macular hole development in which the full thickness of the retinal tissue is completely torn by the tractional force. Moreover, intraoperative finding of macular hole associated with AMD with a possible involvement of subretinal lesion has not been described.

**Case presentation:**

A 78-year-old man diagnosed with wet AMD with subretinal fluid and mild cataract received 8 treatments with intravitreal pegaptanib. After AMD remission, he developed a secondary macular hole in the same eye. He underwent a pars plana vitrectomy that successfully closed the macular hole. Intraoperatively, it was found that the patient’s vitreous was formed and that the ERM and ILM were adherent, suggesting the involvement of a tractional force at the vitreo-retinal interface due to an inflammatory reaction related to AMD and/or intravitreally injected chemical compounds, resulting in macular hole development. Changes in the condition of his AMD and the RPE were observed on a fluorescein angiogram (FA) and an indocyanine green angiogram (IA) that preceded macular hole development, suggesting that subretinal changes may also have been involved in the pathogenesis.

**Conclusion:**

These clinical data, including the intraoperative findings and the temporal changes in the angiograms, suggest that an inflammatory reaction at the vitreo-retinal interface and subretinal lesion related to AMD contribute to the macular hole development in AMD patients treated with intravitreal injection.

## Background

The understanding of macular hole development has been advanced by the knowledge of posterior precortical vitreous pocket formation [1] and recently published studies on surgical findings [2–6]. The tractional force generated at the vitreo-retinal interface is the main factor in macular hole pathogenesis; removal of the internal limiting membrane (ILM) and posterior vitreous, which often exhibits pathological changes, is now a well-accepted surgical approach.

Age-related macular degeneration (AMD) is a leading cause of blindness [7, 8], although recent progress in anti-vascular endothelial growth factor (anti-VEGF) therapy has substantially improved the visual prognosis of one AMD subtype, namely, wet AMD [9–11]. The exudative change in choroidal neovascularization (CNV) can cause pigment epithelial detachment (PED), exudative retinal detachment, and macular edema, whereas contraction of CNV with or without treatment may cause a retinal pigment epithelial (RPE) tear [12]. The exudative change can also cause secondary epiretinal membrane (ERM) formation as a result of a modification of the vitreo-retinal interface [13]. However, macular hole development, which is another disease related to the vitreo-retinal tractional force associated with CNV, is rarely reported. In a macular hole, the tractional force completely tears the full thickness of the retinal tissue. In this report, the clinical course of an AMD case with a macular hole is described.

## Case presentation

**Figure 1:**
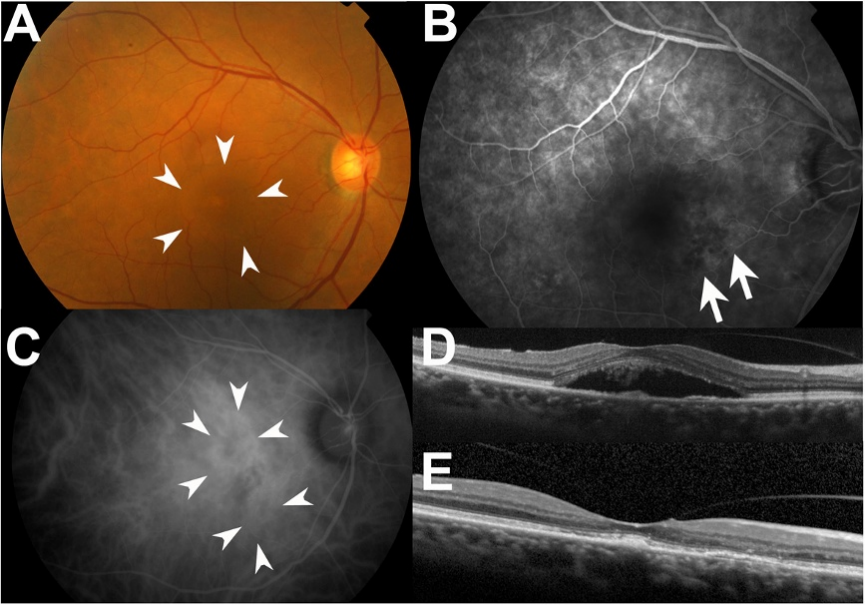
**Macular findings during AMD treatment. (A)** A fundus photograph at his first visit. Subretinal fluid was observed in the macular region (arrowheads). **(B)** A FA obtained prior to the initial intravitreal injection revealed leakage from the CNV of occult with no classic-type (arrows). **(C)** An IA obtained prior to the initial intravitreal injection supported this finding showing the CNV (arrowheads). **(D)** An OCT image prior to the initial intravitreal injection showed subretinal fluid. **(E)** An OCT image at the time of AMD remission after 8 pegaptanib injections. Mild ERM formation and mild vitreo-retinal traction were observed at the fovea.

In August 2009, a 78-year-old man presented with impaired central vision in his right eye and was diagnosed with wet AMD accompanied by subretinal fluid (Figure 1A-D) and mild cataract at the Medical Retina Division Clinic (AMD Clinic) of the Department of Ophthalmology, Keio University Hospital (Tokyo, Japan). His best corrected visual acuity (BCVA) was 0.7 in decimal VA (0.155 in logMAR). He had received eight intravitreal injections of pegaptanib by January 2011, which resolved the exudative changes and improved his BCVA to 1.2 (-0.079). At this time, he had mild ERM formation and mild vitreo-retinal traction at the fovea (Figure 1E), which were not found at his first visit.

**Figure 2:**
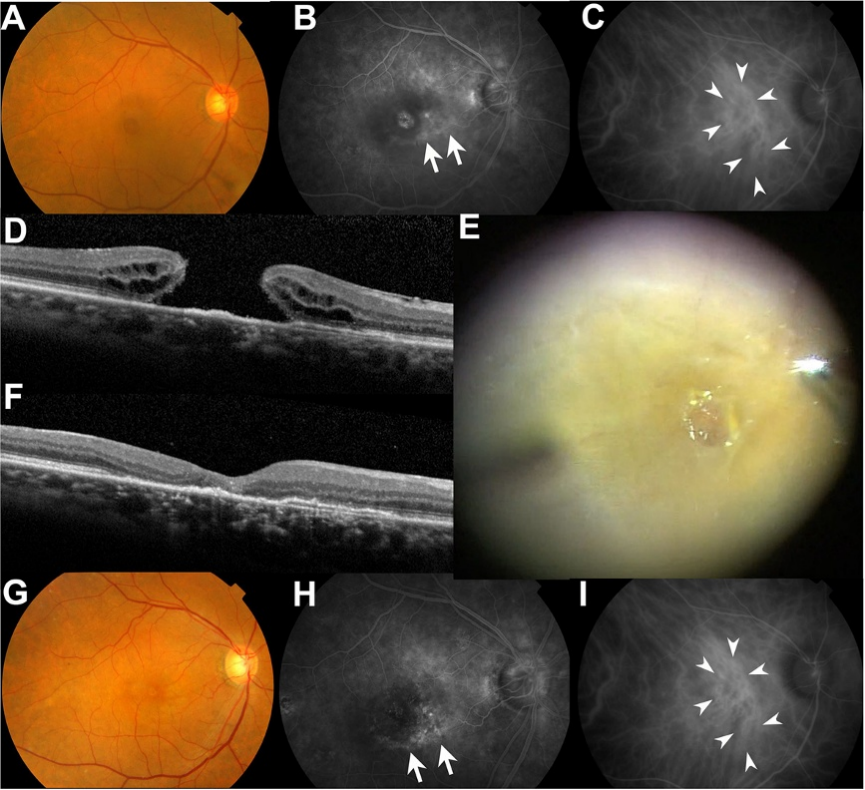
**Macular findings after macular hole formation. (A)** A fundus photograph at the time of macular hole diagnosis. **(B)** A FA obtained after the diagnosis of the macular hole showed enlargement of the hyperfluorescent area (arrows). **(C)** An IA obtained after the diagnosis of the macular hole showed the CNV (arrowheads). **(D)** An OCT image of the macular hole showing an irregularity of the RPE/Bruch complex. **(E)** The intraoperative discovery of an adherent ERM. The macular hole was retracted and distorted by ERM peeling. **(F)** An OCT image after surgery, showing a disrupted photoreceptor cell layer, the ellipsoid zone, and an irregularity of the RPE/Bruch complex after macular hole closure. **(G)** A fundus photograph obtained after macular hole closure. **(H)** Hyperfluorescent area in a FA was clearly observed post-operatively (arrows) after the vitrectomy and the cataract extraction. **(I)** An IA showed that the CNV remained after macular hole surgery and its closure (arrows).

After remission of his exudative change, he suspended his visits to the AMD clinic. However, 2 years later, in February 2013, he had a sudden, substantial impairment of visual acuity. His BCVA was reduced to 0.2 (0.699), which was determined to be due to the formation of a macular hole (Figure 2A-D). A fluorescein angiogram (FA) and an indocyanine green angiogram (IA) showed that the CNV still existed, and the hyperfluorescent area in the FA expanded toward halfway around the fovea (Figure 2B) which may have included atrophic change in the RPE.

In May 2013, he underwent pars plana vitrectomy with gas tamponade (20% SF_6_) and cataract surgery. Intraoperative findings revealed that he had a formed vitreous despite repeated intravitreal injections. Posterior vitreous detachment (PVD) was only observed at the optic disc, and complete PVD was accomplished during the vitrectomy. His posterior hyaloid cortex was thickened, the ERM was sticky (Figure 2E), and the ILM was markedly adherent. The macular hole was retracted and distorted during ERM and ILM peeling. By June 2013, his macular hole had closed and his BCVA had improved to 0.4 (0.398) (Figure 2F,G).

The CNV tissue together with the RPE change secondarily caused by the CNV lesion surrounding the fovea remained post-operatively, as demonstrated by a FA and an IA (Figure 2H,I). The FA image was more clearly recorded post-operatively than pre-operatively (Figure 2B), most likely because of the cataract extraction. Although the macular hole was closed, defects in the ellipsoid zone, which has been called the inner segment/outer segment (IS/OS) line, and irregularities in the photoreceptor layer together with RPE were observed on optical coherence tomography (OCT) (Figure 2F).

## Discussion

There have been no other reports of intraoperative findings of a macular hole developing secondary to wet AMD. Despite several intravitreal injections, the patient’s vitreous was not liquefied. The intraoperative findings included adherent ERM and ILM, which suggested the influence of inflammatory reactions related to the exudative changes of AMD at the vitreo-retinal interface [14]. Moreover, intravitreal injections of a drug can cause intraocular inflammation [11, 15]. The properties of anti-VEGF and its diluents or other included compounds could have also been involved in the inflammatory reaction that caused the modifications of the vitreo-retinal interface. Moreover, the subretinal condition due to AMD preceded macular hole development, suggesting that the pathological RPE may have induced retinal vulnerability, and the CNV might have contracted and accelerated the tangential traction from the subretinal side, and both of which may have possibly contributed to macular hole development.

Although the macular hole was closed, the photoreceptor layer remained disorganized. This disorganization may have resulted because the RPE was in a pathological state due to the AMD and could not induce proper recovery processes during the macular hole closure. The surrounding microenvironment, including the RPE, is important for the proper healing of a macular hole.

One case of macular hole development with AMD after a single injection of intravitreal ranibizumab has been previously reported [16]. In that case, vitreo-macular traction syndrome was observed before treatment and progressed after the injection. However, in the case presented here, there are multiple possibilities for the underlying mechanism of the macular hole development, such as the pathological changes in the subretinal condition including CNV and the RPE, the chronic progression of vitreous modifications due to exudative changes caused by AMD, and intravitreal injections, which cause a sticky ERM and ILM changes.

## Conclusion

These clinical data, including the intraoperative findings and the temporal changes related to AMD, suggest that an inflammatory reaction at the vitreo-retinal interface and subretinal pathological changes contribute to retinal conditions in AMD cases that are treated with intravitreal injections.

## Consent

Written informed consent was obtained from the patient for publication of this case report and any accompanying images. A copy of the written consent is available for review by the editor of this journal.

## Abbreviations

AMD: Age-related macular degeneration
CNV: Choroidal neovascularization
ERM: Epiretinal membrane
FA: Fluorescein angiogram
ILM: Internal limiting membrane
SF_6_: Sulfur hexafluoride.

## References

[1] Kishi S, Shimizu K (1990). Posterior precortical vitreous pocket. Arch Ophthalmol.

[2] Kadonosono K, Itoh N, Uchio E, Nakamura S, Ohno S (2000). Staining of internal limiting membrane in macular hole surgery. Arch Ophthalmol.

[3] Park DW, Sipperley JO, Sneed SR, Dugel PU, Jacobsen J (1999). Macular hole surgery with internal-limiting membrane peeling and intravitreous air. Ophthalmology.

[4] Sakamoto T, Ishibashi T (2009). Visualizing vitreous in vitrectomy by triamcinolone. Graefes Arch Clin Exp Ophthalmol.

[5] Spiteri Cornish K, Lois N, Scott NW, Burr J, Cook J, Boachie C, Tadayoni R, la Cour M, Christensen U, Kwok AK (2014). Vitrectomy with internal limiting membrane peeling versus no peeling for idiopathic full-thickness macular hole. Ophthalmology.

[6] Steel DH, Lotery AJ (2013). Idiopathic vitreomacular traction and macular hole: a comprehensive review of pathophysiology, diagnosis, and treatment. Eye.

[7] Friedman DS, O'Colmain BJ, Munoz B, Tomany SC, McCarty C, de Jong PT, Nemesure B, Mitchell P, Kempen J (2004). Prevalence of age-related macular degeneration in the United States. Arch Ophthalmol.

[8] Gragoudas ES, Adamis AP, Cunningham ET, Feinsod M, Guyer DR (2004). Pegaptanib for neovascular age-related macular degeneration. N Engl J Med.

[9] Brown DM, Kaiser PK, Michels M, Soubrane G, Heier JS, Kim RY, Sy JP, Schneider S (2006). Ranibizumab versus verteporfin for neovascular age-related macular degeneration. N Engl J Med.

[10] Heier JS, Brown DM, Chong V, Korobelnik JF, Kaiser PK, Nguyen QD, Kirchhof B, Ho A, Ogura Y, Yancopoulos GD, Stahl N, Vitti R, Berliner AJ, Soo Y, Anderesi M, Groetzbach G, Sommerauer B, Sandbrink R, Simader C, Schmidt-Erfurth U, VIEW 1 and VIEW 2 Study Groups (2012). Intravitreal aflibercept (VEGF trap-eye) in wet age-related macular degeneration. Ophthalmology.

[11] Rosenfeld PJ, Schwartz SD, Blumenkranz MS, Miller JW, Haller JA, Reimann JD, Greene WL, Shams N (2005). Maximum tolerated dose of a humanized anti-vascular endothelial growth factor antibody fragment for treating neovascular age-related macular degeneration. Ophthalmology.

[12] Chang LK, Sarraf D (2007). Tears of the retinal pigment epithelium: an old problem in a new era. Retina.

[13] Jackson TL, Nicod E, Simpson A, Angelis A, Grimaccia F, Kanavos P (2013). Symptomatic vitreomacular adhesion. Retina.

[14] Funk M, Karl D, Georgopoulos M, Benesch T, Sacu S, Polak K, Zlabinger GJ, Schmidt-Erfurth U (2009). Neovascular age-related macular degeneration: intraocular cytokines and growth factors and the influence of therapy with ranibizumab. Ophthalmology.

[15] Gaudreault J, Fei D, Rusit J, Suboc P, Shiu V (2005). Preclinical pharmacokinetics of Ranibizumab (rhuFabV2) after a single intravitreal administration. Invest Ophthalmol Vis Sci.

[16] Querques G, Souied EH, Soubrane G (2009). Macular hole following intravitrealranibizumab injection for choroidal neovascular membrane caused by age-related macular degeneration. Acta Ophthalmol Scand.

[CR17] The pre-publication history for this paper can be accessed here:http://www.biomedcentral.com/1471-2415/14/114/prepub

